# Constructing machine learning models based on non-contrast CT radiomics to predict hemorrhagic transformation after stoke: a two-center study

**DOI:** 10.3389/fneur.2024.1413795

**Published:** 2024-09-02

**Authors:** Yue Zhang, Gang Xie, Lingfeng Zhang, Junlin Li, Wuli Tang, Danni Wang, Ling Yang, Kang Li

**Affiliations:** ^1^Chongqing Medical University, Chongqing, China; ^2^Department of Radiology, Chongqing General Hospital, Chongqing University, Chongqing, China; ^3^Department of Radiology, Chengdu Third People’s Hospital, Chengdu, China; ^4^North Sichuan Medical College, Nanchong, China

**Keywords:** radiomics, non-contrast computed tomography, machine learning, acute ischemic stroke, hemorrhagic transformation

## Abstract

**Purpose:**

Machine learning (ML) models were constructed according to non-contrast computed tomography (NCCT) images as well as clinical and laboratory information to assess risk stratification for the occurrence of hemorrhagic transformation (HT) in acute ischemic stroke (AIS) patients.

**Methods:**

A retrospective cohort was constructed with 180 AIS patients who were diagnosed at two centers between January 2019 and October 2023 and were followed for HT outcomes. Patients were analyzed for clinical risk factors for developing HT, infarct texture features were extracted from NCCT images, and the radiomics score (Rad-score) was calculated. Then, five ML models were established and evaluated, and the optimal ML algorithm was used to construct the clinical, radiomics, and clinical-radiomics models. Receiver operating characteristic (ROC) curves were used to compare the performance of the three models in predicting HT.

**Results:**

Based on the outcomes of the AIS patients, 104 developed HT, and the remaining 76 had no HT. The HT group consisted of 27 hemorrhagic infarction (HI) and 77 parenchymal-hemorrhage (PH). Patients with HT had a greater neutrophil-to-lymphocyte ratio (NLR), baseline National Institutes of Health Stroke Scale (NIHSS) score, infarct volume, and Rad-score and lower Alberta stroke program early CT score (ASPECTS) (all *p* < 0.01) than patients without HT. The best ML algorithm for building the model was logistic regression. In the training and validation cohorts, the AUC values for the clinical, radiomics, and clinical-radiomics models for predicting HT were 0.829 and 0.876, 0.813 and 0.898, and 0.876 and 0.957, respectively. In subgroup analyses with different treatment modalities, different infarct sizes, and different stroke time windows, the assessment accuracy of the clinical-radiomics model was not statistically meaningful (all *p* > 0.05), with an overall accuracy of 79.5%. Moreover, this model performed reliably in predicting the PH and HI subcategories, with accuracies of 82.9 and 92.9%, respectively.

**Conclusion:**

ML models based on clinical and NCCT radiomics characteristics can be used for early risk evaluation of HT development in AIS patients and show great potential for clinical precision in treatment and prognostic assessment.

## 1 Introduction

Hemorrhagic transformation (HT) is a significant adverse prognosis of acute ischemic stroke (AIS) (1), and AIS is a frequent critical cerebrovascular disease in clinical practice (2). HT is a hemorrhage resulting from the reestablishment of vascular perfusion in the ischemic area after a cerebral infarction. HT is categorized by clinical deterioration and imaging: (1) asymptomatic HT and symptomatic HT according to the presence or absence of clinical exacerbation or a National Institutes of Health Stroke Scale (NIHSS) score ≥4 (3), and (2) parenchymal hemorrhage (PH) and hemorrhagic infarction (HI) according to the European Cooperative Acute Stroke Study-II (ECASS II) (4). HT can occur in AIS patients after perfusion therapy, including endovascular therapy (EVT) and intravenous thrombolysis (IVT), or it can occur naturally during the course of the disease in non-reperfusion-treated patients (5). However, once HT occurs, regardless of the type of HT, the clinical prognosis and functional outcomes are worse (6–8). Therefore, if the risk of developing HT can be predicted early and accurately, clinicians can make optimal treatment decisions and perform more aggressive clinical assessments and monitoring to prevent early clinical deterioration.

Previous studies have shown that clinical-biological indicators (9–12), imaging data (13–16), and clinical features combined with imaging markers (17, 18), such as the NIHSS score, neutrophil-to-lymphocyte ratio (NLR), and hyperdense middle cerebral artery sign (HMCAS), have good predictive performance for HT. Recently, radiomics analysis has emerged as a new technology to assist precision medicine, provide more information about textures that cannot be observed visually, and address the heterogeneity of lesions (19). A recent study (20) alone revealed that radiomic features show promise in quantifying blood-brain barrier disruption. Machine learning (ML), as an important branch of artificial intelligence (AI) that uses various algorithms to focus on learning and training complex data and hyperparameter optimization to build ideal classification, prediction, and evaluation models, is now widely used in the medical field. Several studies have shown that ML can feasibly predict HT (11, 16, 17, 21, 22).

In addition, non-contrast computed tomography (NCCT) remains the imaging test of choice for early identification of ischemic stroke (23) because it is simpler and faster. Therefore, in this study, we wished to validate the correlation between the initial NCCT radiomic features of infarcted brain tissues and the occurrence of HT, while we evaluated multiple ML algorithms based on the clinical and radiomic characteristics retained after screening and selected the optimal ML algorithm to construct the HT prediction models.

## 2 Materials and methods

### 2.1 Patient acquisition and clinical data collection

The research protocol was authorized by the ethics committee of the institution, and the requirement for informed consent was waived.

**Figure 1:**
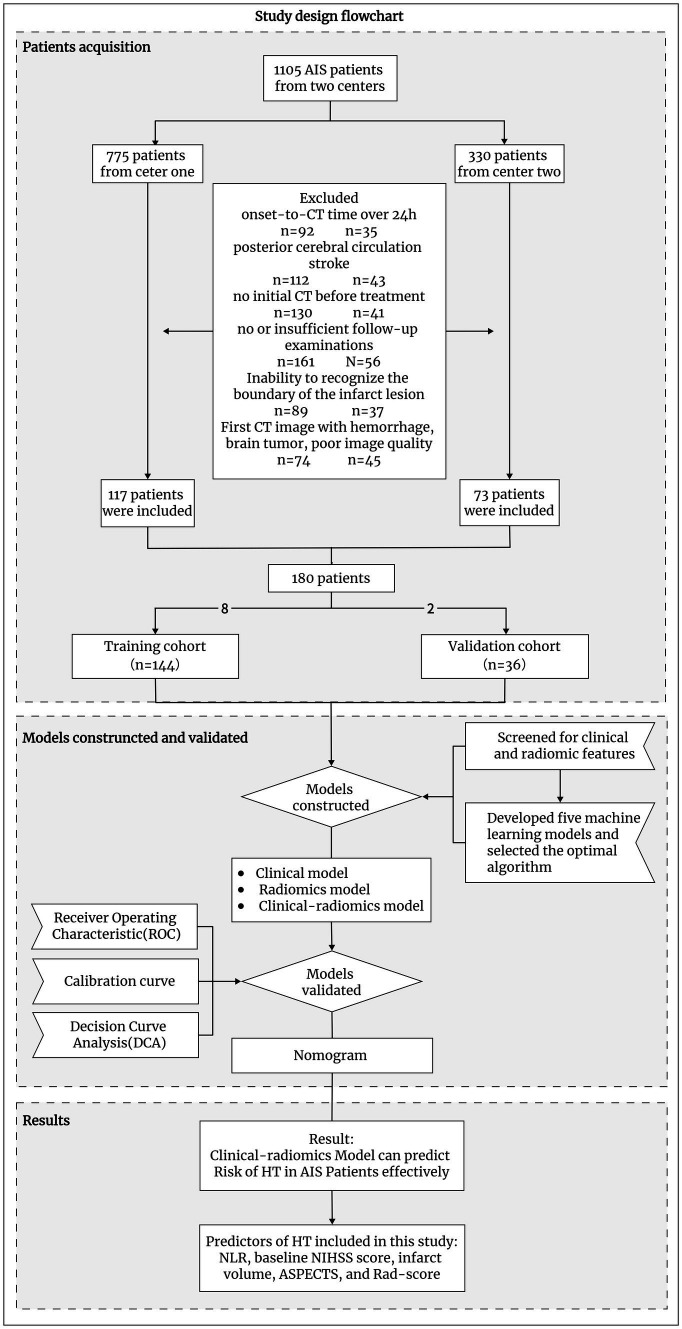
Flow chart of this study design.

We retrospectively enrolled AIS patients who were diagnosed at two centers between January 2019 and October 2023. The inclusion criteria were as follows: (1) stroke patients who had a clear time from symptom onset to the initial non-contrast-enhanced head CT examination of less than 24 h (for patients with wakefulness stroke, we estimated the midpoint between waking and sleeping time as the time of stroke onset); (2) patients with AIS due to anterior circulation large artery occlusion; (3) patients whose image gray values were adjusted, and the CT image could identify patients with infarct region boundaries; otherwise, diffusion-weighted imaging (DWI) or follow-up CT within 6 h was required to match the infarct region to the infarct zone on the initial CT image (18); and (4) CT/magnetic resonance imaging (MRI) follow-up of more than 7 days if HT did not occur within 7 days. Patients with hemorrhagic cerebral infarction on admission, brain tumors, poor quality CT images, or severe missing data were excluded. Ultimately, we included 180 patients (Figure 1).

The clinical data collected included sex, age, smoking status, drinking status, diabetes status, hypertension status, dyslipidemia status, atrial fibrillation status, coagulopathy status, heart failure status, NLR, stroke onset time, baseline NIHSS score (NIHSS_baseline_), and treatment modalities. Clinical data and images were obtained through medical records and a picture archiving communication system (PACS), respectively. We used k-nearest neighbors (KNN) to estimate and supplement some of the missing clinical data to ensure the quality and stability of the data.

### 2.2 Imaging acquisition and analysis

Supplementary Table S1 describes in detail the CT scanner models, scanning parameters, and scanning positions used in this study.

CT images were analyzed individually by two neurological radiologists with 5 years of experience: (1) massive stroke characterized by an infarcted area of more than 1/3 of the area supplied by the middle cerebral artery; (2) HMCAS referred to the increased wall density of the middle cerebral artery on the side of the infarct; (3) the Alberta stroke program early CT score (ASPECTS) was based on the NCCT images to score the ischemic changes of the middle cerebral artery blood supply area in 10 areas of the two levels. Each affected area was subtracted by 1 point, the normal brain CT was 10 points; and (4) HT determined by the presence of hyperdense lesions on follow-up CT images that lasted for more than 2 days or abnormal hyper-density remaining despite removal of residual iodine agent by virtual non-contrast (VNC) of IQon spectroscopy or hemorrhagic signals on follow-up MR images. Agreement between the diagnoses of the two radiologists was judged by kappa analysis, as detailed in Supplementary Table S2. Any discrepancies were resolved by consensus.

### 2.3 Radiomics analysis

After the CT images were anonymized, they were imported in Digital Imaging and Communications in Medicine (DICOM) format into 3D-Slicer software (https://www.slicer.org; version 5.5.0) (24). A single radiologist performed the relevant imaging histological characterization, as shown below.

**Figure 2:**
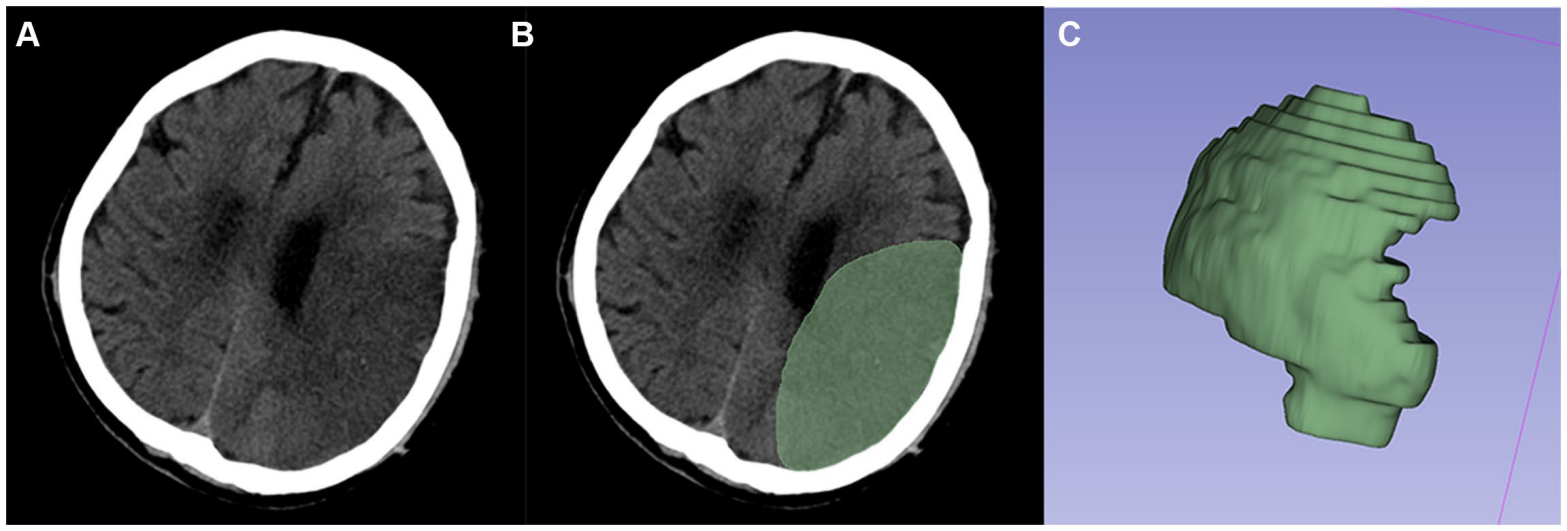
Typical example of ROI segmentation. **(A,B)** Segmented infarcted lesion (green area) on the axial slice of CT images. **(C)** 3D-ROI.

First, the 3D region of interest (ROI) was sketched manually along the contours of the infarcted lesion (Figure 2). If the contours of the lesion cannot be clearly visualized at the first CT examination, the boundary can be drawn with the aid of diffusion-weighted images taken over a short period of time or a second follow-up CT. And the volume of ROIs was measured through this software. Second, to reduce the potential effects of scanner and sweep parameters, we normalized all the ROIs by resampling them via linear interpolation, fixing the image gray values, and smoothing the images via a Gaussian filter. Finally, four major categories of features were extracted for each ROI in this study using the “Radiomics Module” in 3D-Slicer software: first-order statistical features, texture features, morphological features and wavelet features. To ensure the stability of the radiomic features, the above procedure was repeated after 1 month by the same doctor, who randomly selected 90 (1/2) patients from all patients. The intraclass correlation coefficient (ICC) was then calculated based on the radiomics features acquired by the same physician in the ROI sketches drawn before and after two times. Stable features with ICCs above 0.75 were retained, and all the features were normalized using a standard deviation normalization (Z-score) approach.

### 2.4 Feature selection

First, for clinical laboratory data and radiomics features, we screened features for between-group differences by univariate analysis in the training cohort. Then, the least absolute shrinkage and selection operator (LASSO) method was used to removal uncorrelated or redundant radiomic features, and the radiomics features that yielded the optimal predictive efficacy were selected by 5-fold cross-validation. Finally, the logistic regression (LR) classifier algorithm was applied to construct radiomics labels, and the weighting coefficients were summed to calculate the radiomics score (Rad-score).

### 2.5 Evaluating the best ML algorithm for building models

**Figure 3:**
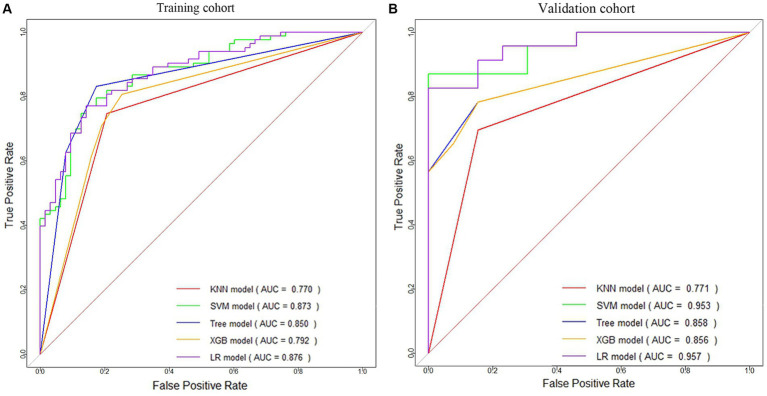
ROC curves of five machine learning models for HT prediction in the training cohort **(A)** and validation cohort **(B)**.

Based on the principle of random sampling in an 8:2 manner, 180 AIS patients were split into a training cohort (*n* = 144, 81 patients with HT) and a validation cohort (*n* = 36, 23 patients with HT). Under the clinical features (*p* < 0.05) and Rad-score obtained from the screening of the above methods, five ML algorithms KNN, support vector machine (SVM), decision tree, extreme gradient boosting (XGB), and LR were developed, and the optimal ML algorithm was determined by comparing their effects to construct a predictive model for HT (Figure 3 and Table 1).

**Table 1:** Performance comparison of five machine learning models for HT prediction.

Model	Group	AUC (95% CI)	Accuracy	Sensitivity	Specificity	PPV	NPV
KNN	Training	0.770 (0.701, 0.839)	0.767	0.827	0.704	0.747	0.794
	Validation	0.771 (0.631, 0.911)	0.750	0.889	0.611	0.696	0.846
SVM	Training	0.873 (0.817, 0.928)	0.795	0.791	0.800	0.868	0.698
	Validation	0.953 (0.892, 1.000)	0.806	0.833	0.750	0.870	0.692
Tree	Training	0.850 (0.790, 0.910)	0.829	0.825	0.831	0.788	0.862
	Validation	0.858 (0.752, 0.964)	0.806	0.846	0.783	0.688	0.900
XGB	Training	0.792 (0.721, 0.862)	0.780	0.746	0.807	0.746	0.807
	Validation	0.856 (0.748, 0.964)	0.806	0.846	0.783	0.688	0.900
LR	Training	0.876 (0.822, 0.931)	0.795	0.798	0.790	0.855	0.714
	Validation	0.957 (0.899, 1.000)	0.861	0.950	0.750	0.826	0.923

HT, hemorrhagic transformation; KNN, k-nearest neighbors; SVM, support vector machine; Tree, decision tree; XGB, extreme gradient boosting; LR, logistic regression; AUC, area under the receiver operating characteristic curve; CI, confidence interval; PPV, positive prediction value; NPV, negative prediction value.

### 2.6 Model construction and evaluation

After determining the optimal ML algorithm, the filtered radiomic features were combined with clinical features to construct the final joint model. In addition, both clinical (constructed by integrating clinical laboratory and traditional imaging features) and radiomics models were constructed for easy comparison. The validation cohort was used to verify the performance of the three models. The area under the receiver operating characteristic curve (AUC) of the constructed predictive models was tested for discrimination by using receiver operating characteristic (ROC) curves, with an AUC closer to 1 suggesting better discrimination of the model; calibration curves were plotted, with a slope closer to 1 suggesting better calibration; clinical applicability was tested by using decision curve analysis (DCA), with a larger area under the decision curve suggesting better clinical applicability of the model; and the DeLong test was used to compare the AUC between the models.

### 2.7 Statistical analysis

The mean ± standard deviation (SD) indicated that the data conformed to a normal distribution, the medians (25% quartile, 75% quartile) indicated that the data did not conform to a normal distribution, and frequencies and constitutive ratios (%) indicated categorical data. Clinical characteristics were assessed using an independent samples *t*-test and a variance test. Intergroup comparisons of the radiomic features were performed using the independent samples *t*-test. The data were analyzed and graphed using R software (version 4.3.1), Python (version 3.9) and SPSS (version 26.0). *p* < 0.05 (two-sided) indicated a significant difference.

## 3 Results

### 3.1 Patients’ clinical features

This study included 180 patients (training cohort 144, test cohort 36) from two centers—95 males, 85 females, and patients aged 42–94 years with a median age of 70.51 years (62.00, 79.25). Among them, 104 patients developed HT (76 with PH and 28 with HI), and 76 patients had no HT. All the data were not significantly different between the training and validation cohorts (*p* > 0.05) (Table 2).

**Table 2:** Characteristics of patients in the training and validation cohorts.

Variables	All (*n* = 180)	Training cohort (*n* = 144)	Validation cohort (*n* = 36)	*p*
Sex				0.724
Female	85 (47.22%)	73 (50.69%)	12 (33.33%)	
Male	95 (52.78%)	71 (49.31%)	24 (66.67%)	
Age group, year				0.853
≤50	10 (5.56%)	8 (5.56%)	2 (5.56%)	
50–60	28 (15.56%)	23 (15.97%)	5 (13.89%)	
60–70	49 (27.22%)	40 (27.78%)	9 (25.0%)	
70–80	55 (30.56%)	45 (31.25%)	10 (27.78%)	
>80	38 (21.11%)	28 (19.44%)	10 (27.78%)	
Age, year	70.51 (62.0, 79.25)	70.13 (62.0, 79.0)	72.03 (64.0, 81.25)	0.395
Smoking				0.691
No	126 (70.0%)	101 (70.14%)	25 (69.44%)	
Yes	54 (30.0%)	43 (29.86%)	11 (30.56%)	
Drinking				0.159
No	131 (72.78%)	107 (74.31%)	24 (66.67%)	
Yes	49 (27.22%)	37 (25.69%)	12 (33.33%)	
Diabetes				1.000
No	139 (77.22%)	115 (79.86%)	24 (66.67%)	
Yes	41 (22.78%)	29 (20.14%)	12 (33.33%)	
Hypertension				0.767
No	59 (32.78%)	45 (31.25%)	14 (38.89%)	
Yes	121 (67.22%)	99 (68.75%)	22 (61.11%)	
Hyperlipidemia				1.000
No	113 (62.78%)	92 (63.89%)	21 (58.33%)	
Yes	67 (37.22%)	52 (36.11%)	15 (41.67%)	
Atrial fibrillation				0.540
No	91 (50.56%)	67 (46.53%)	24 (66.67%)	
Yes	89 (49.44%)	77 (53.47%)	12 (33.33%)	
Coagulopathy				0.406
No	131 (72.78%)	106 (73.61%)	25 (69.44%)	
Yes	49 (27.22%)	38 (26.39%)	11 (30.56%)	
Heart failure				1.000
No	79 (43.89%)	63 (43.75%)	16 (44.44%)	
Yes	101 (56.11%)	81 (56.25%)	20 (55.56%)	
NLR	8.13 (3.83, 9.97)	8.47 (3.92, 10.2)	6.8 (3.37, 7.42)	0.207
Stroke onset Time, h	7.88 (3.0, 10.0)	8.11 (3.0, 10.0)	6.93 (3.0, 9.0)	0.358
NIHSS_baseline_	14.48 (11.0, 18.0)	14.58 (11.0, 18.0)	14.08 (9.75, 18.0)	0.670
Treatment modalities				0.243
Non-reperfusion	97 (53.89%)	78 (54.17%)	19 (52.78%)	
IVT	36 (20.0%)	25 (17.36%)	11 (30.56%)	
EVT	29 (16.11%)	24 (16.67%)	5 (13.89%)	
IVT with EVT	18 (10.0%)	17 (11.81%)	1 (2.78%)	
Massive stroke				0.562
No	83 (46.11%)	66 (45.83%)	17 (47.22%)	
Yes	97 (53.89%)	78 (54.17%)	19 (52.78%)	
HMCAS				0.848
No	100 (55.56%)	78 (54.17%)	22 (61.11%)	
Yes	80 (44.44%)	66 (45.83%)	14 (38.89%)	
ASPECTS	4.47 (2.0, 7.0)	4.46 (2.0, 7.0)	4.5 (2.0, 7.25)	0.941
Infraction volume, cm^3^	116.92 (37.97, 165.16)	118.1 (40.78, 164.06)	112.23 (28.73, 171.44)	0.758

Measurement data are expressed as median (25% quartile, 75% quartile); categorical data are expressed in number (%); NLR, neutrophil-to-lymphocyte ratio; NIHSS_baseline_, Baseline National Institutes of Health Stroke Scale score; IVT, intravenous thrombolysis; EVT, endovascular therapy; HMCAS: hyperdense middle cerebral artery sign; ASPECTS, Alberta stroke program early CT score.

Clinical and imaging characteristics with component differences associated with HT were screened by univariate analysis in the training cohort (Table 3). We found that the NLR, baseline NIHSS score, infarct volume and Rad-score were greater and the ASPECTS was lower in the HT group than in the no-HT group (all *p* < 0.01).

**Table 3:** Univariate analysis of characteristics in the training cohort.

Variables	HT (*n* = 81) (56.25%)	No-HT (*n* = 63) (43.75%)	*p*
Sex			0.710
Female	40 (49.38%)	33 (52.38%)	
Male	41 (50.62%)	30 (47.62%)	
Age group, year			0.592
<50	4 (4.94%)	4 (6.35%)	
50–60	15 (18.52%)	8 (12.7%)	
60–70	18 (22.22%)	22 (34.92%)	
70–80	26 (32.1%)	19 (30.16%)	
>80	18 (22.22%)	10 (15.87%)	
Age, year	70.51 (62.0, 79.0)	69.65 (62.0, 79.0)	0.667
Smoking			0.584
No	55 (67.9%)	46 (73.02%)	
Yes	26 (32.1%)	17 (26.98%)	
Drinking			1.000
No	58 (71.6%)	49 (77.78%)	
Yes	23 (28.4%)	14 (22.22%)	
Diabetes			1.000
No	65 (80.25%)	50 (79.37%)	
Yes	16 (19.75%)	13 (20.63%)	
Hypertension			0.377
No	25 (30.86%)	20 (31.75%)	
Yes	56 (69.14%)	43 (68.25%)	
Hyperlipidemia			0.141
No	55 (67.9%)	37 (58.73%)	
Yes	26 (32.1%)	26 (41.27%)	
Atrial fibrillation			1.000
No	34 (41.98%)	33 (52.38%)	
Yes	47 (58.02%)	30 (47.62%)	
Coagulopathy			0.123
No	58 (71.6%)	48 (76.19%)	
Yes	23 (28.4%)	15 (23.81%)	
Heart failure			0.451
No	29 (35.8%)	34 (53.97%)	
Yes	52 (64.2%)	29 (46.03%)	
NLR	9.78 (4.76, 11.3)	6.77 (3.51, 9.32)	0.011^*^
Stroke onset Time, h	8.04 (4.0, 10.0)	8.2 (3.0, 10.0)	0.896
NIHSS_baseline_	16.28 (13.0, 19.0)	12.39 (9.0, 15.5)	<0.001^*^
Treatment modalities			0.218
Non-reperfusion	45 (55.56%)	33 (52.38%)	
IVT	12 (14.81%)	13 (20.63%)	
EVT	10 (12.35%)	14 (22.22%)	
IVT with EVT	14 (17.28%)	3 (4.76%)	
Massive cerebral stroke			0.103
No	21 (25.93%)	45 (71.43%)	
Yes	60 (74.07%)	18 (28.57%)	
HMCAS			0.853
No	32 (39.51%)	46 (73.02%)	
Yes	49 (60.49%)	17 (26.98%)	
ASPECTS	3.1 (1.0, 5.0)	6.21 (5.0, 8.0)	<0.001^*^
Infraction volume, cm^3^	158.71 (88.71, 213.0)	65.88 (17.0, 106.71)	<0.001^*^

^*^p < 0.05; measurement data are expressed as median (25% quartile, 75% quartile); categorical data are expressed in number (%); NLR, neutrophil-to-lymphocyte ratio; NIHSS_baseline_, Baseline National Institutes of Health Stroke Scale score; IVT, intravenous thrombolysis; EVT, endovascular therapy; HMCAS: hyperdense middle cerebral artery sign; ASPECTS, Alberta stroke program early CT score.

### 3.2 Radiomics score

**Figure 4:**
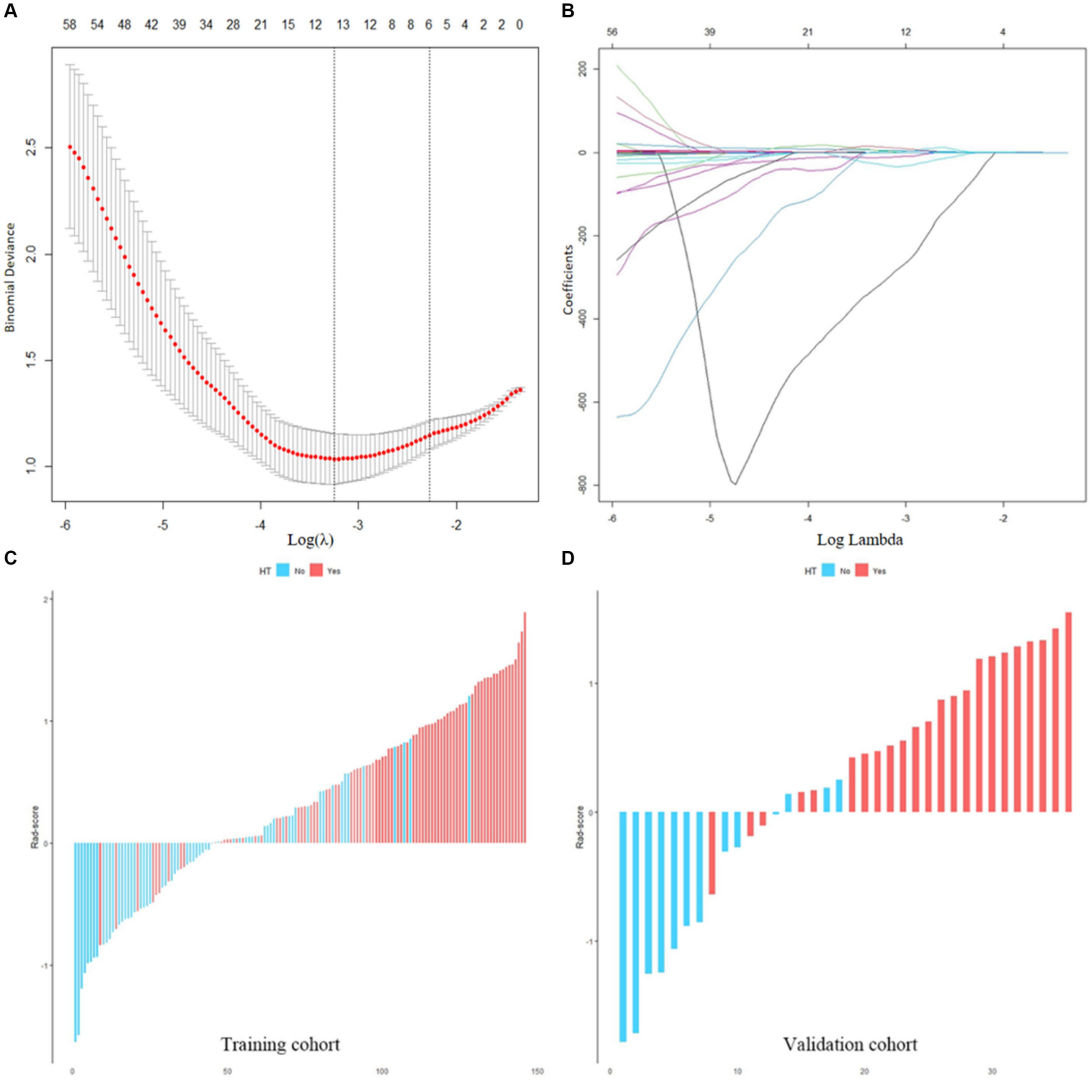
**(A,B)** Screening of radiomics features. **(C,D)** Waterfall plots of the Rad-score in the training and validation cohorts. Red is set to HT, blue is set to non-HT.

A total of 851 radiomics features were calculated from the initial CT images of each patient. (1) A total of 706 features were obtained after ICC screening, (2) 182 features were screened via *t*-tests, and (3) six features highly correlated with HT were screened by the LASSO regression downscaling method and 5-fold cross-validation (Figures 4A,B). The six features were analyzed using LR and Rad-score was calculated as shown in equation.


$$f\left(x\right)=\sum \beta_{i}x_{i}+\beta_{0}$$

$\beta_{i}$ was the coefficient of each feature, $x_{i}$ was the specific feature, and $\beta_{0}$ was the constant (Supplementary Table S3). Figures 4C,D shows the Rad-score of each patient in both cohorts for further analysis. The results indicated that Rad-scores were greater and significantly different in HT patients than in non-HT patients (mean 0.693 vs. −0.266, *p* < 0.001).

### 3.3 Performances of the models

**Figure 5:**
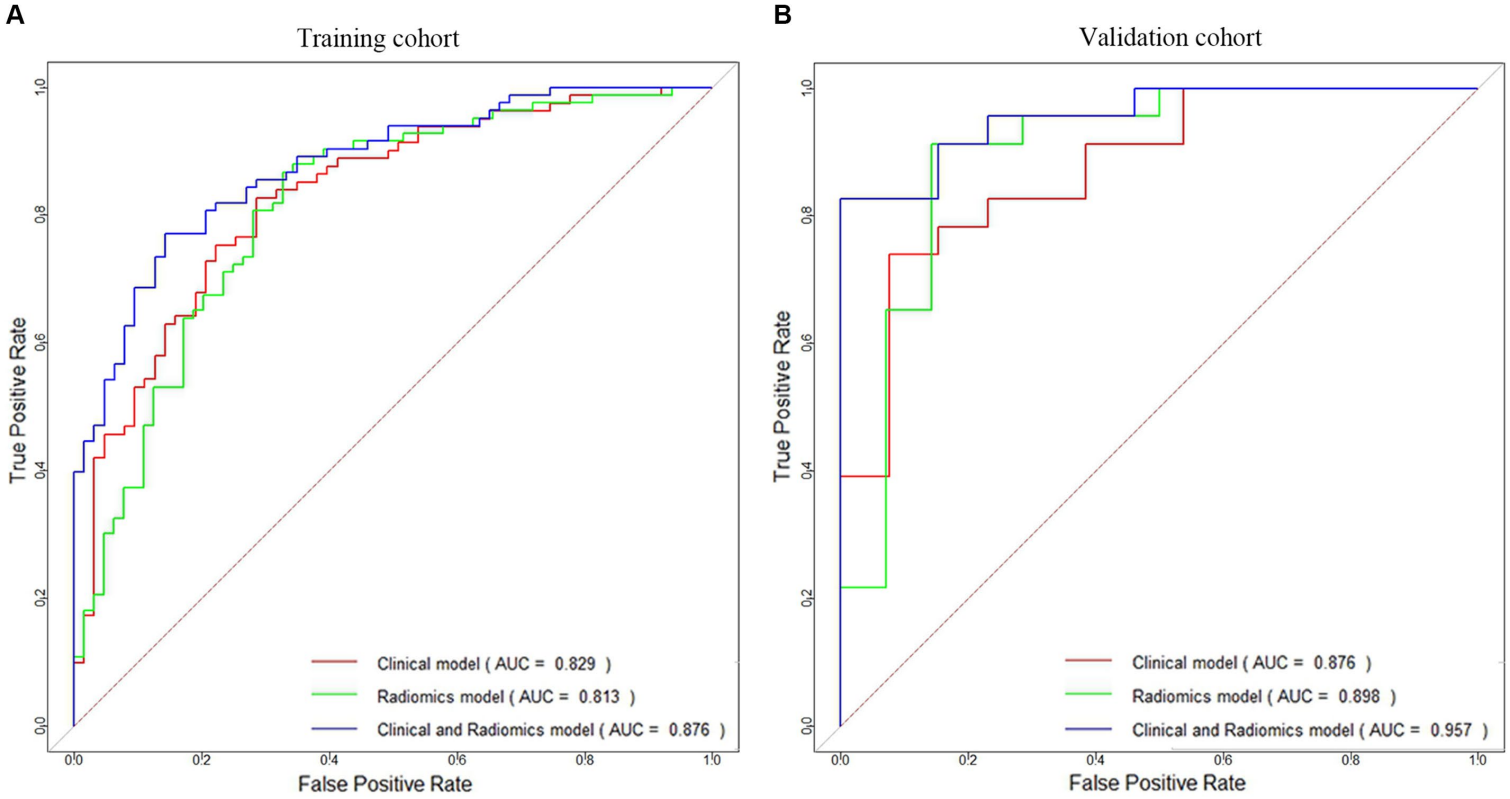
ROC curves of the clinical model, radiomics model, and clinical-radiomics model for HT prediction constructed in the training cohort **(A)** and validation cohort **(B)**.

This study revealed that the LR algorithm performed the most well. Therefore, this study established multiple logistic regression prediction models according to the histologic and clinical features obtained from the above screening, namely, the clinical model, the radiomics model, and the clinical-radiomics fusion model. In the training and validation cohorts, the AUCs were 0.829 (95% CI 0.762–0.896) and 0.876 (95% CI 0.759–0.994) for the clinical model and 0.813 (95% CI 0.742–0.884) and 0.898 (95% CI 0.776–1.000) for the radiomics model, respectively, and the AUCs of the clinical-radiomics model were 0.876 (95% CI 0.822–0.931) and 0.957 (95% CI 0.899–1.000), respectively (Figures 5A,B).

Table 4 compares the results of the three models, and Supplementary Figure S1 shows the DeLong test results. The results indicated that although the difference in predictive ability between the three models was not significant in the training and validation cohorts (*p* > 0.05), the predictive ability of the clinical-radiomics model improved in both cohorts when clinical and radiological histological features were analyzed together.

**Table 4:** Performance of the clinical model, radiomics model, clinical-radiomics model for HT prediction.

Model	Group	AUC (95% CI)	Accuracy	Sensitivity	Specificity	PPV	NPV
Clinical model	Training	0.829 (0.762, 0.896)	0.771	0.786	0.750	0.815	0.714
	Validation	0.876 (0.759, 0.994)	0.806	0.900	0.688	0.783	0.846
Radiomics model	Training	0.813 (0.742, 0.884)	0.755	0.764	0.741	0.819	0.672
	Validation	0.898 (0.776, 1.000)	0.865	0.910	0.800	0.870	0.857
Clinical-radiomics model	Training	0.876 (0.822, 0.931)	0.795	0.798	0.790	0.855	0.714
	Validation	0.957 (0.899, 1.000)	0.861	0.950	0.750	0.826	0.923

HT, hemorrhagic transformation; AUC, area under the receiver operating characteristic curve; CI, confidence interval; PPV, positive prediction value; NPV, negative prediction value.

**Figure 6:**
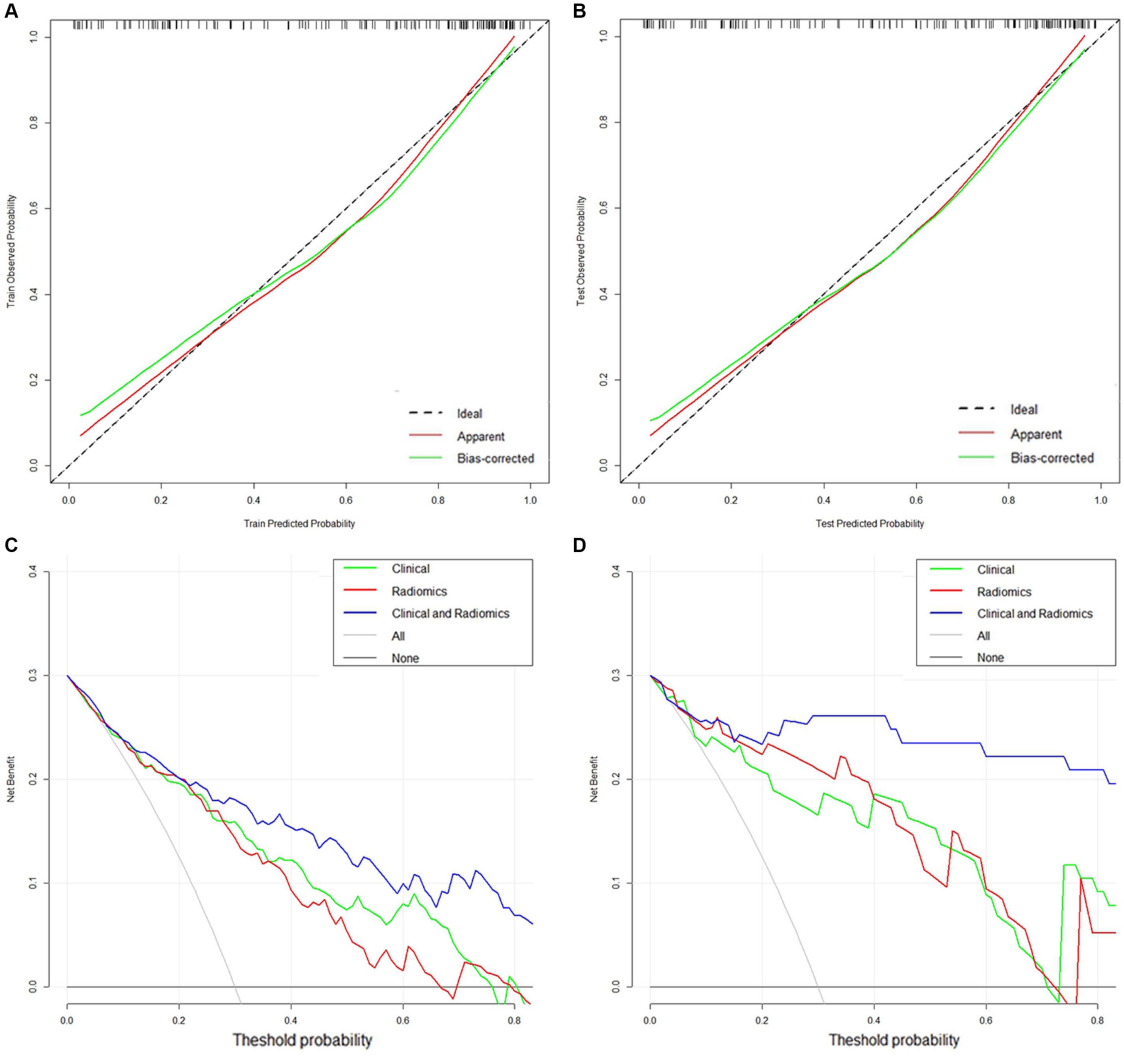
**(A,B)** Calibration curves of the clinical-radiomics model in the training and validation cohorts. **(C,D)** DCA curves of the clinical model, radiomics model, and clinical-radiomics model in the training and validation cohorts.

In addition, the calibration curves showed high concordance between the predicted and actual risk for the clinical-radiomics models in both cohorts (Figures 6A,B). The DCA curves indicated that the clinical-radiomics fusion model had a greater net clinical benefit advantage than the single model (Figures 6C,D).

**Figure 7:**
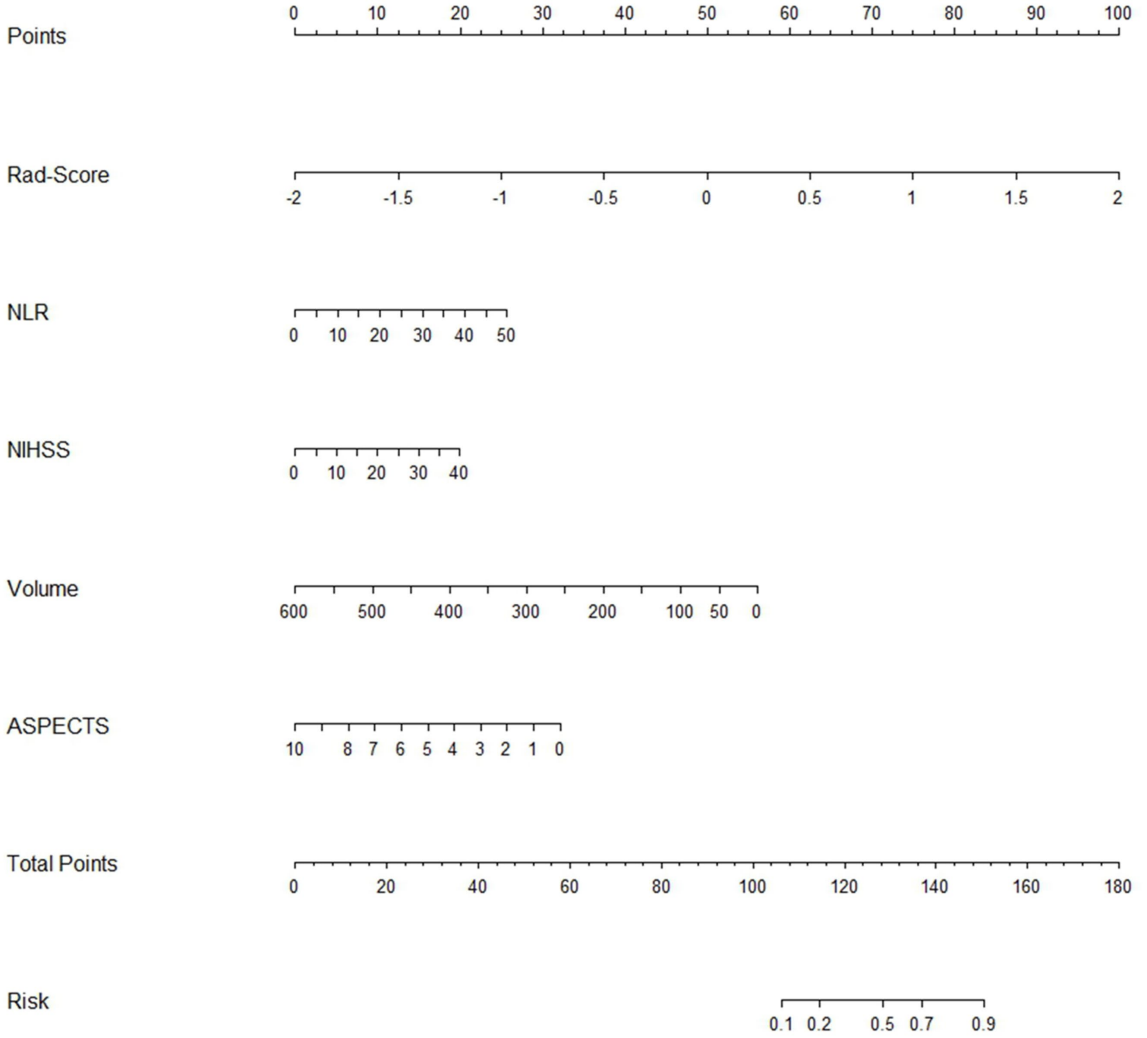
The clinical-radiomics nomogram for predicting HT after stroke.

Finally, we transformed the multifactorial logistic regression model built using the Rad-score in conjunction with clinical characteristics into a visualized nomogram, which made the predictive model readable and facilitated the assessment of patients (Figure 7).

**Figure 8:**
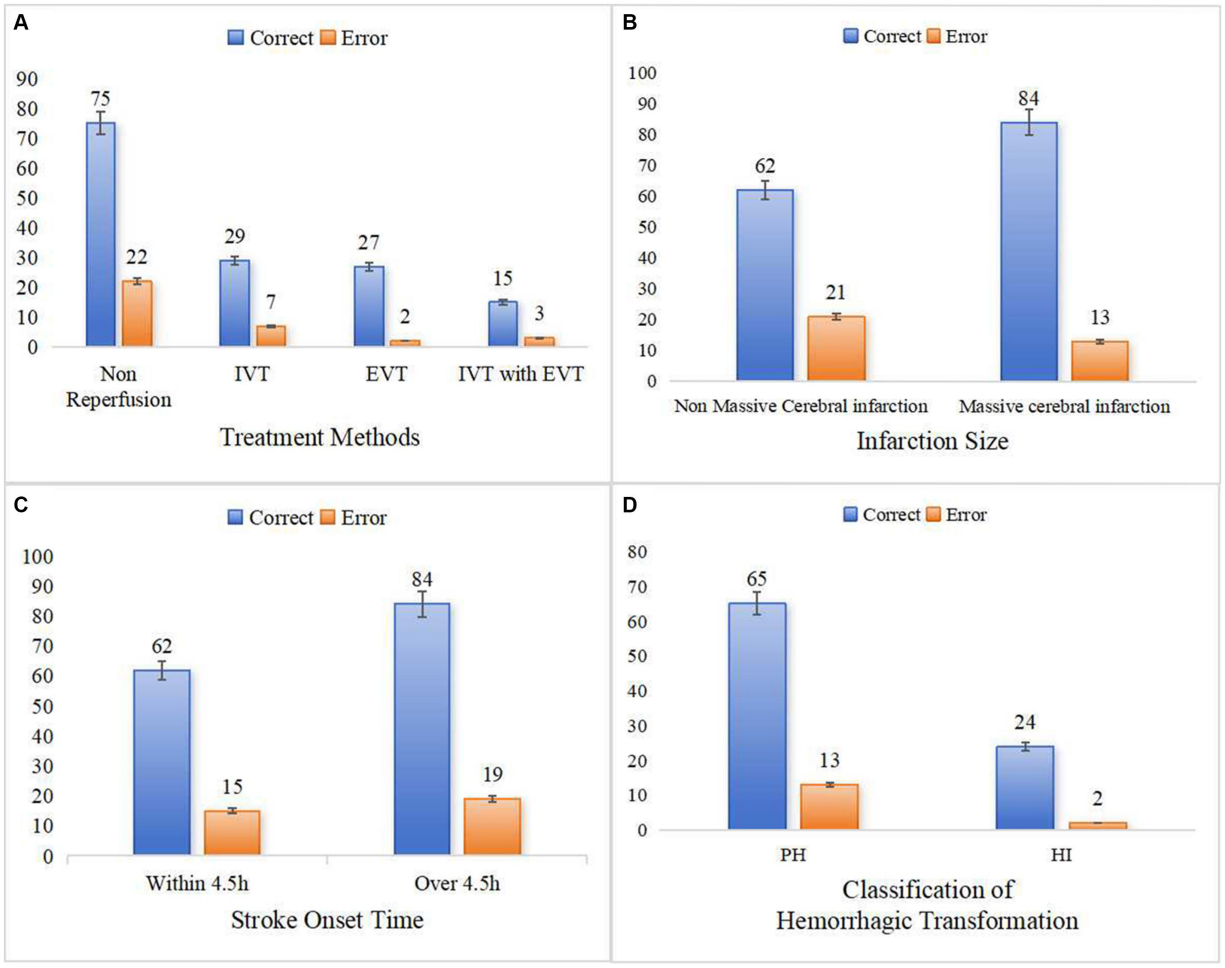
Predictive ability of clinical-radiomics model in different subgroups. **(A)** Shows the different treatment methods. **(B)** Shows the different infarction size. **(C)** Shows the different stroke onset time. **(D)** Shows the two classifications of HT.

This study further analyzed and discussed the expression ability of the clinical-radiomics model in different subgroups. The model predicted HT with an accuracy of 77.3–93.1% for stroke patients receiving different treatment modalities (Figure 8A). The accuracy of the model in predicting HT was 86.6 and 74.7% for patients with massive stroke and non-massive stroke, respectively (Figure 8B). In addition, the model predicted HT in patients within 4.5 h and over 4.5 h after stroke onset with accuracies of 80.5 and 81.6%, respectively (Figure 8C). We found that none of the model predictions were significantly different in the different subgroup analyses (all *p* > 0.05). Furthermore, this clinical-radiomics model performed reliably in predicting the PH and HI subcategories, with accuracies of 82.9 and 92.9%, respectively. In short, the model predicted HT well, with accuracies of 79.5 and 95.0% in the training and validation cohorts, respectively (Figure 8D).

## 4 Discussion

HT is a significant adverse prognostic factor for AIS and determines patients’ therapeutic decisions and functional prognosis. Therefore, in this study, we explored the risk factors for HT by combining clinical laboratory indices and imaging images of stroke patients, constructed imaging biomarkers for predicting HT, and finally developed and validated a reliable predictive model for HT.

As in previous studies, this study revealed that four clinical characteristics, the NLR (12, 25, 26), baseline NIHSS score (27–29), infarct volume (30–32), and ASPECTS (33, 34), were significant risk factors strongly associated with HT. Neutrophils are important indicators of inflammation, which is an important factor in secondary brain injury after stroke (35, 36). At the same time, the systemic immunosuppression that occurs after stroke causes a decrease in lymphocytes (37), the relative decrease in lymphocytes in response to acute physiological stress promotes an increase in inflammatory cytokines, which exacerbates ischemic injury (38, 39). Therefore, a high NLR reflects the degree of brain damage after stroke and is important for predicting HT. Increasing values of the NIHSS score, which is used as an index to assess neurologic damage in stroke patients, tend to indicate more severe blood-brain barrier disruption. Consistent with previous findings (27–29), our study suggested that higher baseline NIHSS scores are associated with a greater risk of HT. In addition, this study revealed that larger infarct volumes and lower ASPECTS were more likely to lead to HT because massive stroke can lead to more severe cytotoxic edema due to ischemia and hypoxia, exacerbating the breakdown of the blood-brain barrier (30, 31). Therefore, if the admission of a patient with AIS reveals a low ASPECTS and a large infarct volume, the physician needs to be alerted to the possibility of a high risk of HT in this patient when laboratory results are unavailable.

Radiomics features are more focused on internal differences within the infarct area than are clinical laboratory markers reflecting systemic systems and imaging markers simply reflecting the extent of the infarct. Thus, in the present study, the radiomic features of the infarcted area of the first CT on admission were also obtained. Six optimal radiomic features were ultimately included through screening, including one gray-level dependence matrix (GLDM) feature reflecting the small correlation of lower gray values in the infarct region, two shape features describing the size and flatness of the cerebral infarcts, one gray-level run length matrix (GLRLM) feature reflecting the running variance of the image, and two GLRLM features measuring the distribution of the long stroke. Finally, the radiomics model constructed by combining these features predicted HT well (AUC = 0.898 for the validation cohort). The DCA curves indicate that clinical models (constructed by integrating clinical laboratory and traditional imaging features) and radiomics models have essentially the same benefits in clinical applications. This finding suggested that radiomics features can be used to effectively and adequately assess the status of blood-brain barrier damage by indirectly identifying microscopic differences within the infarct zone from changes in image voxels alone while ignoring global immune status and imaging changes visualized by the naked eye (40). It is worth noting that in order to reduce the differences in imaging between different devices, and considering the clinical applicability of the model, all images were standardized in this study, and these operations may have a certain impact on the extraction of radiomics features. Therefore, when using the model, ensuring that all input images are uniformly preprocessed can fully utilize the best effect of the model.

In contrast to previous studies that constructed models to predict HT based only on clinical or radiomic characteristics, this study constructed a clinical-radiomics model that demonstrated strong predictive performance in the validation cohort (AUC of 0.957, 95% accuracy), while the DCA curves indicated that the model had a greater net clinical benefit advantage in HT prediction than did the clinical and radiomics models. In addition, the model showed greater potential for subgroup prediction with different treatment methods, different infarct sizes, and different stroke time windows, as well as for HT classification prediction, reflecting the greater stability and robustness of the model in different environments. Moreover, only a few studies (15, 41) have focused on the risk of HT in AIS patients not receiving recanalization therapy, who account for the majority of AIS patients. In fact, the need to develop individualized treatment for patients who cannot receive recanalization therapy is also increasing. Therefore, the present study simultaneously included stroke patients who received different treatment modalities, such as IVT, EVT, bridging therapy, and non-reperfusion therapy, constructing a model with a wider range of applicability. Finally, visualizing the model with a nomogram also makes the application easier and can quickly assist neurologists in comprehensively assessing patients’ HT risk and customizing personalized treatment plans, resulting in a greater rate of patient benefit.

However, there are several limitations. First, although the study was a two-center study, the sample size was small, and the study was retrospective. Second, the limitations of CT image resolution can lead to errors in sketching the boundary of the infarct lesion. Moreover, some patients with hyperacute AIS for whom the infarction boundary could not be determined on the first CT images and for whom follow-up CT/MRI images were not acquired within 6 h were not included in the study. Therefore, the reproducibility and clinical feasibility of this study were somewhat limited. Finally, because some laboratory data were incomplete, we did not include some important predictors associated with HT, such as matrix metalloproteinase (MMP) (42). There was also some information that was not analyzed in detail, such as coagulation function, which was not divided into fibrin degradation products (FDPs) and D-dimer. In future studies, big data support, multicenter validation, multidisciplinary collaboration, and prospective evaluation may help us gradually move closer to the goal of precision medicine.

## 5 Conclusion

In conclusion, the ML model constructed on the basis of initial NCCT images and clinical characteristics of AIS patients is valuable for early clinical screening of high-risk groups prone to HT and for the development of individualized and precise prevention and treatment measures.

## Data Availability

The raw data supporting the conclusions of this article will be made available by the authors, without undue reservation.

## Glossary

AIS: Acute ischemic stroke
HT: Hemorrhagic transformation
HI: Hemorrhagic infarction
PH: Parenchymal hemorrhage
NIHSS: National Institutes of Health Stroke Scale
NIHSS_baseline_: Baseline NIHSS score
ECASS II: European Cooperative Acute Stroke Study-II
EVT: Endovascular therapy
IVT: Intravenous thrombolysis
NLR: Neutrophil-to-lymphocyte ratio
HMCAS: Hyperdense middle cerebral artery sign
ML: Machine learning
AI: Artificial intelligence
NCCT: Non-contrast computed tomography
DWI: Diffusion-weighted imaging
MRI: Magnetic resonance imaging
KNN: k-nearest neighbors
ASPECTS: Alberta stroke program early CT score
Rad-score: Radiomics score
ROI: Region of interest
ICC: Intraclass correlation coefficient
LASSO: Least absolute shrinkage and selection operator
LR: Logistic regression
SVM: Support vector machine
XGB: Extreme gradient boosting
ROC: Receiver operating characteristic
AUC: Area under the receiver operating characteristic curve
ACC: Accuracy
CI: Confidence interval
PPV: Positive prediction value
NPV: Negative prediction value
DCA: Decision curve analysis
GLDM: Gray-level dependence matrix
GLRLM: Gray-level run length matrix
MMP: Matrix metalloproteinase
FDPs: Fibrin degradation products
