# Supplementary material for: Constructing machine learning models based on non-contrast CT radiomics to predict hemorrhagic transformation after stoke: a two-center study
Source: Front Neurol. 2024 Sep 2;15:1413795. doi: 10.3389/fneur.2024.1413795 (PMC11402658; doi:10.3389/fneur.2024.1413795)
Supplement: Supplementary file 1 [file Data_Sheet_1.docx]

Supplementary Material

**TABLE S1** Models of CT scanners and scanning parameters for two centers.

| **Center** | **Equipment** | **Tube voltage (KV)** | **Tube current (mAs)** | **Matrix size** | **Slice thickness(mm)** | **Slice-Spacing (mm)** |
| --- | --- | --- | --- | --- | --- | --- |
| 1 | IQon Spectral CT* (Philips) | 120 | 220 | 512×512 | 5 | 5 |
| 1, 2 | LightSpeed VCT (GE)* | 120 | 220 | 512×512 | 5 | 5 |

*The scanning position was supine, with the orbital-nasal tract as the scanning baseline. The scanning area was from the top of the head to the base of the skull.

**TABLE S2** Agreement analysis of interpretation of imaging findings.

| **Image analysis** | **Kappa value** | ***P* value** |
| --- | --- | --- |
| Massive stroke | 0.845 | <0.001 |
| HMCAS | 0.865 | <0.001 |
| ASPECTS | 0.808 | <0.001 |
| HT | 0.989 | <0.001 |
| Classification of HT | 0.892 | <0.001 |

HMCAS = Hyper-dense middle cerebral artery sign; ASPECTS = Alberta stroke program early CT score; HT = Hemorrhagic transformation.

**TABLE S3** Names and coefficients of six radiomics features.

| **Numbers** | **Features(**$x_{i}$**)** | **Coefficient(**$\beta_{i}$**)** |
| --- | --- | --- |
| f1 | `wavelet-LHH_gldm_SmallDependenceLowGrayLevelEmphasis` | -53.92688543 |
| f2 | original_shape_Flatness | 0.47242119 |
| f3 | `wavelet-HLL_glrlm_RunVariance` | 0.28975783 |
| f4 | `wavelet-LHH_glrlm_LongRunEmphasis` | 0.21865867 |
| f5 | `wavelet-LHL_glrlm_LongRunEmphasis` | 0.05393468 |
| f6 | original_shape_Maximum2DDiameterColumn | 0.00532634 |

Rad-score is the sum of the products of the retained features and their respective corresponding coefficients.


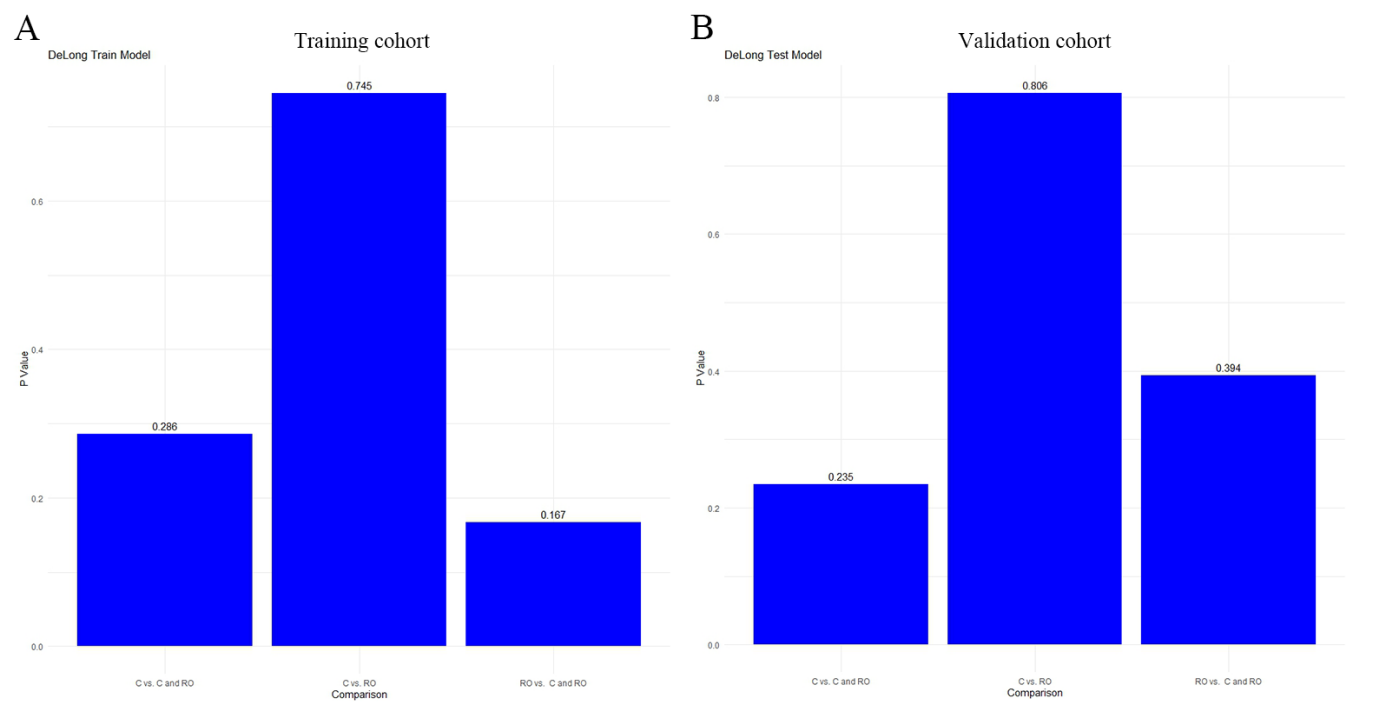


**FIGURE S1**

DeLong test for three models in training and validation cohorts. C, Clinical model; RO, Radiomics model; C and RO, Clinical-radiomics model.
